# Assessment of Oncology Advanced Practice Professional Willingness to Participate in Medical Aid in Dying

**DOI:** 10.1001/jamanetworkopen.2022.39068

**Published:** 2022-10-26

**Authors:** Jonathan Singer, Courtney Daum, Megan J. Shen, Gabrielle Zecha, Louise Kaplan, Kathy Plakovic, Meagan Blazey, Molly Arnold, Barbara Silko, Kelsey Baker, Elizabeth T. Loggers

**Affiliations:** 1 Department of Psychological Sciences, Texas Tech University, Lubbock; 2 Clinical Research Division, Fred Hutchinson Cancer Center, Seattle, Washington; 3 Division of Oncology, University of Washington, Seattle; 4 College of Nursing, Washington State University, Vancouver; 5 Fred Hutchinson Cancer Center, Seattle, Washington; 6 Clinical Research Division, Department of Clinical Biostatistics, Fred Hutchinson Cancer Center, Seattle, Washington

## Abstract

**Importance:**

In 2021, New Mexico passed legislation allowing nurse practitioners and physician assistants (referred to herein as advanced practice professionals [APPs]) to prescribe medications for medical aid in dying (MAID). Other US states with existing MAID laws (eg, Washington) are also considering expanding MAID prescribing authority to APPs. There is a lack of research exploring APP knowledge of, willingness to, and comfort with acting as a prescribing or consulting clinician for MAID.

**Objective:**

To assess perspectives of oncology APPs regarding MAID, including their willingness to prescribe and/or consult for MAID and factors associated with willingness.

**Design, Setting, and Participants:**

This cross-sectional, self-report survey study used data collected from APPs working at a comprehensive cancer center in Washington State in fall 2021.

**Main Outcomes and Measures:**

The primary survey question was whether APPs would be willing to participate in death with dignity, the term used for MAID in Washington. Survey questions evaluated how influential specific factors were on APP views on MAID as well as respondents’ knowledge of and comfort with aspects of the MAID process.

**Results:**

Of 167 eligible APPs, 77 (46.1%) responded to the survey. Most respondents (68 [88.3%]) reported their race and ethnicity as White; 72 (93.5%) identified as a woman. Medical oncology (28 [36.4%]) was the most common field of practice, and 21 respondents (27.3%) reported having practiced as an APP for 6 to 10 years. Of all respondents, 61 (79.2%) reported having at least 1 patient who inquired about MAID; depending on the question, less than a third of respondents (5.0%-27.0%) endorsed feeling knowledgeable or very knowledgeable about any aspect of the MAID process. In this study, 39 APPs (50.6%) endorsed being willing to participate in MAID either as a consulting or prescribing clinician, whereas 31 (40.3%) were uncertain of whether they would participate. Willingness to participate was associated with having had more patients pursue MAID (33 of the 39 willing participants [84.6%] vs 15 of the 31 unsure participants [48.4%] reported having 1 or more patients pursue MAID). Higher knowledge and comfort scores were both significantly associated with increased odds of being willing to participate (odds ratio, 1.14 per 1-point score increase [95% CI, 1.03-1.27]; *P* = .01) vs unsure (1.18 [95% CI, 1.07-1.30; *P* = .001).

**Conclusions and Relevance:**

The results of this survey study suggest that oncology APPs may require preparation for the addition of MAID to their scope of practice. This study also raises questions for future research regarding support for APPs who may be considering participation in MAID but question their role or want physician involvement.

## Key Points

**Question:**

Are nurse practitioners and physician assistants (advanced practice professionals [APPs]) willing to prescribe medications for medical aid in dying (MAID), and are they knowledgeable about and comfortable with prescribing medication or consulting for MAID?

**Findings:**

In this survey study of 77 APPs at a comprehensive cancer center in Washington State, 51% reported that they were willing to prescribe and/or consult for MAID. However, depending on the question, 43% or fewer reported being comfortable or very comfortable and 27% or fewer reported being knowledgeable or very knowledgeable regarding behaviors integral to MAID participation.

**Meaning:**

Advanced practice professionals may require preparation, including training, for the addition of MAID to their scope of practice.

## Teaser

This survey study uses self-reported response data from advanced practice professionals (ie, nurse practitioners and physician assistants) in a single Washington State cancer center to assess their willingness to participate in prescribing or consulting for medical aid in dying.

## Introduction

In the US, medical aid in dying (MAID) allows patients with a terminal illness and a life expectancy of less than 6 months to request a prescription for oral medication that will end their life upon self-administration.^1^ On April 8, 2021, New Mexico Governor Michelle Lujan Grisham signed the Elizabeth Whitefield End-of-Life Options Act, making it the 10th US state and 11th US jurisdiction to permit MAID.^2^ This law is the first in the US to allow physician assistants (PAs) and nurses licensed in advanced practice (herein NPs, acknowledging that titles, credentials, and actual roles for advanced nursing professionals vary by state but typically include nurse practitioners, nurse anesthetists, clinical nurse specialists, and nurse midwives) to prescribe and consult on MAID cases. Although access to MAID has increased both in the US and worldwide to approximately 200 million people,^3^ Canada has historically been the only country that has allowed NPs to prescribe MAID medications within some provinces,^4^ and there is little to no existing research. Similar legislation to expand MAID consulting and prescribing authority to PAs and NPs (referred to collectively herein as advanced practice professionals [APPs]) was considered in both 2021 and 2022 in Washington State, where physician participation in MAID via the Death with Dignity Act (DWD)^5^ has been legal since 2009. Although the legislative amendment did not pass, these actions highlight the need for research in this area, particularly to understand APP knowledge about, comfort with, and willingness to adopt this new behavior. Knowledge, comfort, and willingness affect both APP prescribing practices and the provision of evidence-based care for patients.^6,7,8^ Prior studies also looked at these factors in relation to advance care planning and hospice referrals^9^ as well as other potentially controversial topics such as cannabis use^10^ and HIV preexposure prophylaxis (PrEP).^11,12,13,14^ Finally, it is important to understand to what extent existing concerns about physician participation in MAID—such as inaccuracy of prognostication, conflict with professional norms and roles, and potential worsening of preexisting social and cultural biases against older adults and individuals with terminal illness—apply to APP participation.^15,16^

Although the American Medical Association now includes ethical arguments both for and against MAID, its formal stance remains one of opposition to physician participation.^17^ Yet from 1998 to 2015, 336 physicians in Oregon and Washington wrote 1545 MAID prescriptions, averaging 3.4 total prescriptions (range, 1-71) each.^18^ In contrast with the American Medical Association’s position, the American Academy of Hospice and Palliative Medicine’s stance is now one of studied neutrality.^19^ In a comprehensive review of the issue, the American Academy of Physician Assistants (AAPA) stated the following:

AAPA does not advocate assisted suicide. However, AAPA feels that the ethical, compassionate, well-intentioned provider who discusses voluntary self-termination of life by competent informed terminally ill patients is not to be subject to prosecution. PAs are front line caregivers for the dying. They should take a leadership role in educating… regarding the need for enlightened and progressive policies in this area. AAPA believes that the most effective way to minimize the issue of assisted suicide is to optimize care and maximize quality of life for patients at the end of life.^17^

Finally, the American Association of Nurse Practitioners, to our knowledge, does not have a policy addressing MAID.

Regardless of these position differences on MAID, APPs actively participate in the care of individuals who are seriously ill and dying. In fact, APPs may be disproportionately represented in advance care planning, including the completion of forms such as the Medical Order for Life-Sustaining Treatment and the Physician (or Portable) Order for Life-Sustaining Treatment. A previous study reported that NPs in West Virginia completed almost twice as many forms, on average, as physicians and that a substantially greater proportion of those forms indicated “do not resuscitate” and “comfort measures.”^20^ This finding may have been attributable at least in part to nearly 4-fold more NPs practicing in palliative care compared with physicians. However, in another study in Oregon, 11.9% of all forms in 2015 were completed by NPs.^21^ Furthermore, at least 7 of the 10 states that legalized MAID also allow APPs to sign Physician (or Portable) Order for Life-Sustaining Treatment forms, pointing to the important role of APPs in these states.^21^

We aimed to assess the perspectives of oncology APPs regarding MAID, including their willingness to prescribe and/or consult in MAID cases and factors associated with willingness. The opinion of oncology practitioners is important because from 2009 to 2017, 76.4% of participants who requested MAID in Oregon and Washington had cancer as their underlying terminal illness.^22^ Results from this study may potentially inform the New Mexico experience, enrich policy debates within Washington, and enlighten future discussions regarding APP participation, training, and educational needs regarding MAID.

## Methods

This survey study was approved by the Fred Hutchinson Cancer Center (FHCC) Institutional Review Board, which included a waiver of documentation of consent. This study followed the American Association for Public Opinion Research (AAPOR) reporting guideline, specifically on internet surveys of named participants.

### Study Sample and Design

This cross-sectional descriptive study used self-report survey methodology. The potential sample included all APPs working at FHCC between August 8 and September 8, 2021, and identified via an existing institutional email list.

### Study Variables

Research materials defined DWD for participants to (1) prevent confusion with euthanasia and (2) ensure consistent understanding of its current legality. Further, Washington State legislation refers to 2 distinct clinician roles: consulting and attending (physician). In this survey, we used the terms *consulting* and *prescribing* to highlight prescribing as one of the distinct differences in the responsibility of these 2 clinician roles. Because no validated or reliable survey measures exist, they were developed by all study investigators (including 4 doctoral-trained investigators with extensive experience in survey creation and testing) using psychometric best practices^23^ and reviewed for content and face validity. The primary question related to APP willingness to participate in DWD was based on participants’ response to the following: “If it were legal for APPs to participate as consulting or prescribing clinicians, would you plan on participating in Death With Dignity, in any capacity?” Demographic data were collected for age, gender, race and ethnicity, religion, and years in APP practice. Gender and race and ethnicity were predefined (eAppendix in the Supplement) and were of interest because we hypothesized that most FHCC APPs would identify as women and White. Participants could select all applicable categories or mark “other” to self-describe. Questions about APPs’ personal experience with DWD asked for the number of patients who had inquired and/or pursued DWD. Seven questions evaluated how influential specific factors were toward APP views on DWD, 6 questions asked about their DWD knowledge, and 7 questions asked about their comfort with aspects of the DWD process; all questions used a 5-point Likert scale. Questions addressing respondent comfort and knowledge are commonly found in the clinician survey literature.^10,11,12,13,14,24,25,26^

### Procedures

Potential participants were emailed a short description of the study and a link to respond to a 1-time, online Research Electronic Data Capture–based survey (eAppendix in the Supplement); they then read an institutional review board–approved study description and opted in or out. Potential survey participants were contacted every 10 days, for a maximum of 4 recruitment attempts.

### Statistical Analysis

Responses are summarized using means (±SDs) for continuous variables and frequencies with percentages for categorical responses. Fisher exact tests and 2-sample *t* tests were used to evaluate associations of APP willingness to participate in DWD and their clinical experience, sociodemographic factors, and self-reported knowledge and comfort. We collapsed 2 “yes” subgroups (“as consulting and prescribing” and “as consulting but not prescribing”) into 1 group (“willing to participate in some capacity”) and compared this group with those who answered “unsure” or “no” because (1) the primary rationale for inclusion of APPs in MAID is to improve access, which is achieved if a clinician participates in either role; and (2) the majority of APPs were willing to participate in both roles (discussed in the Results). Participants who answered no to the willingness question were excluded from regression analyses because of the small sample size. Univariable logistic regression was used to investigate the association of participant willingness (willing to participate in some capacity vs unsure) and their cumulative knowledge and comfort scores. Statistical significance was based on a 2-sided α of .05. No correction was made for multiple comparisons. Quantitative data analyses were performed (K.B.) using SAS software (version 9.4).

Content analysis was performed by 2 experienced qualitative researchers (J.S. and E.T.L.). Coding of free text provided in response to a single, voluntary follow-up question (“Please tell us why”) was conducted independently and then combined via a consensus process consistent with methods described by Cascio et al.^27^ All codes identified across 2 or more subjects are presented.

## Results

### Sociodemographic Factors and Prior Experience With Death With Dignity

Of 167 APPs eligible for this survey study, 77 (46.1%) responded. All email addresses were valid. Ninety-two potential participants opened the email (55.1%), and 15 opted out (16.3%; the response rate among those who opened the email was 83.7%). All questions were voluntary and missing data were generally limited (specifically, 2 questions with 2 nonrespondents and 10 questions with 1 nonrespondent) except for age (16 nonrespondents [20.8%]).

The mean (SD) participant age was 40.4 (9.5) years. Of all respondents, 68 (88.3%) were White and 72 (93.5%) identified as women. Medical oncology (28 [36.4%]) was the most common field of practice, and 21 respondents (27.3%) reported practicing as APPs for 6 to 10 years. Of all respondents, 42 (54.5%) reported prior personal and/or professional experience with DWD, whereas 30 (39.0%) reported prior educational activities (eg, didactics, grand rounds) on this topic. In addition, 61 APPs (79.2%) reported having had at least 1 patient inquire about DWD, whereas 35 (45.5%) reported having 4 or more such patients. Finally, 52 respondents (67.5%) reported working with at least 1 patient who actively pursued DWD.

### Willingness of APPs to Participate in Death With Dignity

With regard to willingness to participate in DWD, 39 APPs (50.6%) endorsed willingness to participate in some capacity, 31 (40.3%) were unsure, and 7 (9.1%) denied any level of willingness to participate. Of those willing to participate in some capacity, 29 (74.4% [37.7% of all participants]) reported being willing to both prescribe and consult, whereas 10 (25.6% [13.0% of all participants]) reported only willingness to consult. Overall, there were no statistically significant differences in sociodemographic factors or number of prior patients inquiring for willing participants vs unsure participants (Table 1). However, there was an association between reported number of patients pursuing MAID and willingness to participate, with 33 of the 39 willing participants [84.6%] vs 15 of the 31 unsure participants [48.4%] reporting 1 or more patients pursuing DWD (*P* = .01).

**Table 1.** Sociodemographic and Clinical Characteristics of Advanced Practice Professionals Who Were Either Unsure or Willing to Participate in Medical Aid in Dying (MAID) in Some Capacity (Either as a Consulting or Prescribing Clinician or Both)

Characteristic	No. of survey respondents (%)^a^		*P* value^b^
	Willing to participate in some capacity (n = 39)	Unsure (n = 31)	
Age, y, mean (SD)^c^	39.9 (9.8)	40.3 (9.1)	.89
Gender			
Women	35 (89.7)	30 (96.8)	.37
Men	4 (10.3)	1 (3.2)	
Race and ethnicity^d^			
Racial or ethnic minority^e^	4 (10.5)	2 (6.7)	.69
White	34 (89.5)	28 (93.3)	
Religion^f^			
Agnostic, atheist, or none	20 (52.6)	18 (58.1)	.22
Christian	11 (28.9)	11 (35.5)	
Jewish or other	5 (13.2)	0	
Spiritual	2 (5.3)	2 (6.5)	
Years in APP practice			
0-2	8 (20.5)	4 (12.9)	.89
3-5	6 (15.4)	6 (19.4)	
6-10	11 (28.2)	8 (25.8)	
11-20	9 (23.1)	7 (22.6)	
>20	5 (12.8)	6 (19.4)	
Field			
Hematology or malignant hematology	8 (20.5)	4 (12.9)	.10
Transplant or immunotherapy	6 (15.4)	12 (38.7)	
Medical oncology	18 (46.2)	8 (25.8)	
Supportive care, surgical oncology, radiation, or other	7 (17.9)	7 (22.6)	
Personal experience with serious illness			
None	5 (12.8)	4 (12.9)	>.99
Personal	1 (2.6)	1 (3.2)	
Family, loved one, or friend	29 (74.4)	23 (74.2)	
Both	4 (10.3)	3 (9.7)	
Personal experience with MAID			
None	14 (35.9)	16 (51.6)	.54
Family, loved one, or friend	1 (2.6)	0	
Professional experience	22 (56.4)	14 (45.2)	
Both	2 (5.1)	1 (3.2)	
No. of patients who inquired about MAID			
0	7 (17.9)	8 (25.8)	.21
1-3	10 (25.6)	12 (38.7)	
≥4	22 (56.4)	11 (35.5)	
No. of patients who pursued MAID^g^			
0	6 (15.4)	15 (50.0)	.01
1-3	18 (46.2)	9 (30.0)	
≥4	15 (38.5)	6 (20.0)	

^a^ Excludes 7 advanced practice professionals who indicated that they would not participate in any capacity.

^b^ *P* values were calculated with the *t* test or Fisher exact test for count data.

^c^ Data missing for 10 individuals for the willing subgroup and 6 individuals for the unsure subgroup.

^d^ Data missing for 1 individual for the willing and unsure subgroups.

^e^ Race and ethnicity is not further delineated due to the small sample size.

^f^ Data missing for 1 individual for the willing subgroup.

^g^ Data missing for 1 individual for the unsure subgroup.

**Figure 1.**
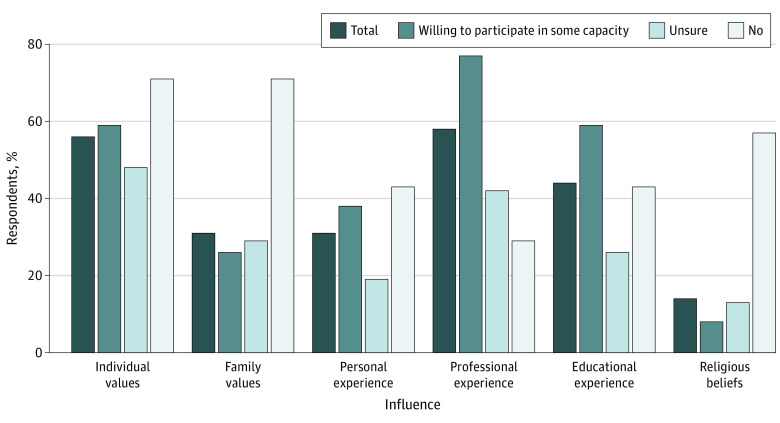
Advanced Practice Professionals Reporting That Certain Values and/or Experiences Are Very Influential or Extremely Influential to Their View on Medical Aid in Dying, by Willingness to Participate

Willing and unsure APPs often rated professional experience (43 [61.4%]), individual values (38 [54.3%]), and educational experience (31 [44.3%]) as “very influential” or “extremely influential” in their views regarding DWD. Based on Fisher exact tests, professional experience (*P* = .004) and educational experience (*P* = .008) were reported as significantly more influential by those willing to participate in some capacity compared with those who were unsure (Figure 1).

The content analysis included 59 of 77 respondents (76.6%) who provided additional text. The results revealed that the 2 most common responses identified among willing APPs were (1) supporting patient choices and values and (2) providing all options to patients (5 respondents each; Table 2). In contrast, the most common response among the unsure was the need for training, information, and education (10 respondents).

**Table 2.** Responses From the Content Analysis of Open-Ended Text From 59 of 77 Advanced Practice Professionals by Willingness to Participate in Medical Aid in Dying

Willingness to participate (No. of respondents)	Themes (No. of times endorsed)	Representative quote
No (7 of 7)	Not comfortable (2)	“Based off of my beliefs, I would not feel comfortable prescribing a medication.”
	Against beliefs (5)	
	Moral issues with DWD (6)	
Unsure (26 of 31)	Not comfortable (6)	“I believe hospice services can be really useful in end-of-life care and am unsure when I would suggest Death With Dignity over hospice.”
	Moral issues with DWD (2)	
	Not relevant to [their] work (3)	
	Need training, information, and education (10)	
	Wanting physician to sign off/not [their] role (6)	
	Other options for end-of-life care (2)	
Yes, as consulting but not prescribing (5 of 10)	Need training, information, and education (2)	“Would need more training before deciding”
	Wanting physician to sign off/not [their] role (2)	
	Support patient’s choice and values (3)	
	Providing all options to the patient (2)	
Yes, as consulting and prescribing (21 of 29)	Need training, information, and education (3)	“I believe patient[s] need to be involved in care. Therefore, patients should be involved in their death. Death With Dignity is one avenue for patients to take control of death. My job is to give patient[s] options and not to judge.”
	Support patient’s choice and values (5)	
	APPs spend most of the time with patients/have a better connection with patient (2)	
	Providing all options to the patient (3)	

Abbreviations: APP, advanced practice professional; DWD, death with dignity.

### Attitudes Toward, Knowledge of, and Comfort With Medical Aid in Dying

Although 70 respondents (90.9%) agreed or strongly agreed that MAID should be legal, these views varied by willingness to participate. Among those who were willing or unsure, 67 (95.7%) agreed or strongly agreed that MAID should be legal vs 3 (42.9%) who were unwilling to participate. When participants were asked whether APPs should be able to act in a consulting role or in a prescribing role, 63 (81.8%) and 47 (61.0%) agreed or strongly agreed, respectively. Specifically, 61 (87.1%) and 46 (65.7%) of willing or unsure respondents endorsed APPs acting as consulting and prescribing clinicians, respectively, compared with 2 (28.6%) and 1 (14.3%) of those unwilling to participate.

**Figure 2.**
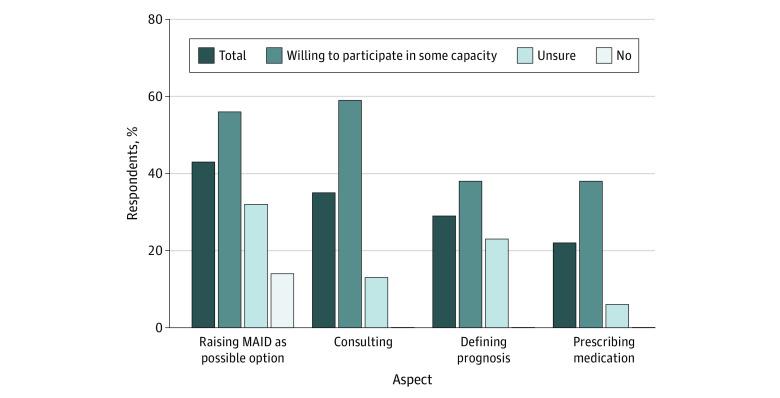
Advanced Practice Professionals Reporting Feeling Comfortable or Very Comfortable With Aspects of the Medical Aid in Dying (MAID) Process, by Willingness to Participate

**Figure 3.**
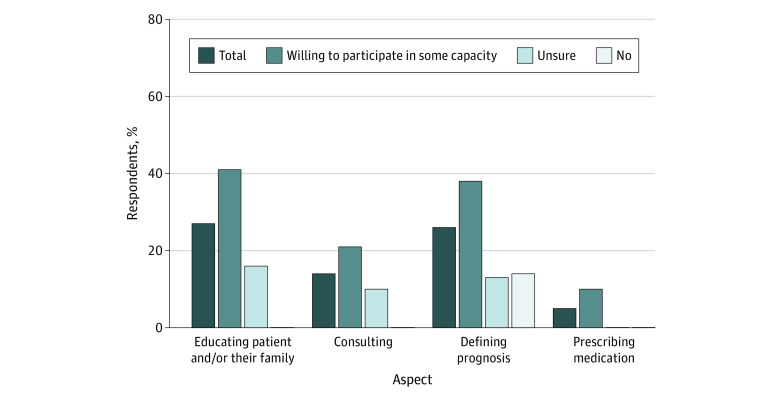
Advanced Practice Professionals Reporting Feeling Knowledgeable or Very Knowledgeable With Aspects of the Medical Aid in Dying Process, by Willingness to Participate

Participant knowledge about and comfort with MAID was low. Depending on the question, less than a third of all respondents (5.0%-28.6%) endorsed feeling knowledgeable or very knowledgeable about specific aspects of the MAID process. Similarly, one-quarter to one-half of all respondents (22.0%-43.0%) reported feeling comfortable or very comfortable with specific aspects of MAID. However, willing participants reported greater knowledge and comfort regarding MAID compared with their unsure or unwilling colleagues (Figure 2 and Figure 3).

### Factors Associated With Willingness to Participate in Medical Aid in Dying

In univariable logistic regression models, higher cumulative knowledge scores (OR per 1-point increase in score, 1.14 [95% CI, 1.03-1.27]; *P* = .01) and cumulative comfort scores (1.18 [95% CI, 1.07-1.30]; *P* = .001) were both associated with increased odds of being willing in some capacity (vs unsure) to participate in MAID. Because knowledge and comfort scores were highly correlated, they could not be included in a single logistic regression model.

## Discussion

In this survey study, 50.6% of APPs indicated that they would be willing to participate in MAID either as a consulting or prescribing clinician if legally allowed. This finding is in agreement with surveys of US physician attitudes toward and willingness to participate in MAID in states where it is legal.^28,29^ For example, Ganzini et al^29^ reported that 59.0% of physicians felt MAID was not unethical or immoral and 51.0% supported or strongly supported legalization of MAID, including 53.0% of physicians who would consider MAID for themselves if they had a terminal illness. Furthermore, physicians reported being willing to act in a consultant role and in a prescribing role 46.3% and 28.1% to 33.0% of the time, respectively.

In this study, most survey respondents who were willing to participate in MAID in some capacity cited the importance of prior education and professional experience and a sense that supporting this option for individual patients is important. Our results suggest that willingness to participate was also associated with having 1 or more patients pursue MAID. Half of APPs were willing to participate despite relatively high rates of self-reported discomfort and lack of knowledge as well as scant prior education or training on MAID. The other half were either unwilling or uncertain of their willingness. In our exploration of why 40.3% of respondents were unsure about participating, we observed that clinical or sociodemographic factors did not seem to play an important role. However, APPs pointed to a desire for further education and physician support—and, to quote one participant, “I need time to process this.”

These results seem to be consistent with the experience of NPs when prescribing of schedule II to IV controlled substances was added to their scope of practice.^30^ A qualitative study revealed that legalization of controlled substance prescribing was insufficient to promote the adoption of this role for some NPs.^30^ Other studies have also demonstrated a difference between positive attitudes toward advance care planning, level of knowledge, and self-reported behaviors to support end-of-life patient options among APPs and physicians.^9,31,32^

Prior studies of physicians have also reported a tension between what clinicians can or feel they ought to do and their individual knowledge, comfort, willingness, and actual clinical behavior in other domains.^11,13^ For instance, in a sample of Minnesota physicians, 58.1% felt that cannabis was a legitimate medical treatment but 50.0% did not feel ready or did not want to answer patient questions about cannabis.^10^ Moore et al^11^ found that a majority of clinicians felt they were obligated to provide information and to prescribe HIV PrEP, as well as to refer a patient if they could not prescribe, despite low rates of prescribing. Authors have found that clinicians who have prescribed PrEP are more comfortable taking a sexual history, more knowledgeable about PrEP, and more likely to have had a patient request it.^11,12^ Similarly, with respect to MAID, physicians have reported greater willingness than preparedness to participate.^28^ In a previous study, having been asked to prescribe a lethal prescription was associated with the number of patients with a terminal illness that the clinician had cared for recently as well as clinician willingness to prescribe, finding care of patients with a terminal illness intellectually satisfying, and improving one’s knowledge of pain medications since MAID legalization.^29^ Our results also underscore the important role of patients’ requests and personal clinician attitudes with respect to APPs and MAID.

Finally, this study also raises questions about the best way to support APPs who may be considering participation in MAID but question their role or want physician support. This issue may be particularly salient in rural areas, where the goal of such legislation is to improve patient access, but may leave APPs with little clinician support. In addition, although NPs are often independent practitioners, PAs require a collaborative agreement with a physician. Therefore, PAs may be limited by the willingness of the collaborating physician to participate in MAID.

### Limitations

This survey study has limitations. First, the results are limited to a moderate-sized sample of relatively homogenous oncology APPs from a single institution in urban Washington. As a result, we were not able to investigate certain valuable comparisons (eg, across race and ethnicity or rurality). Second, the characteristics and perspectives of nonrespondents are unknown. Third, although DWD was globally defined for participants and we used the descriptive label of “prescribing” clinician to distinguish between consulting and attending physician roles, it is unknown whether the lack of a more complete definition of each role affected participant responses in a meaningful way. Additionally, only 7 APPs in this study were unwilling to participate in any capacity, which limits our insights into their perspective. It is also unknown whether the observed distribution of willingness is representative beyond the single institution.

## Conclusions

In this survey study of oncology APPs, our results suggest that although there may be a pool of APPs willing to participate in MAID, there is also a need for additional education and training about MAID. This need likely exists even among APPs with extensive experience with patients with terminal illness. Finally, it will be important to conduct a larger, longitudinal study assessing APP participation in MAID and related outcomes. A longitudinal study could also assess whether and what type of education and training is effective in supporting APPs as they decide whether to adopt this scope of practice.

## Notes

Supplement. **eAppendix.** Study Information Sheet and Research Electronic Data Capture Survey Data Dictionary
