# Supplementary material for: Assessment of Oncology Advanced Practice Professional Willingness to Participate in Medical Aid in Dying
Source: JAMA Netw Open. 2022 Oct 26;5(10):e2239068. doi: 10.1001/jamanetworkopen.2022.39068 (PMC9606841; doi:10.1001/jamanetworkopen.2022.39068)
Supplement: Supplement. — eAppendix. Study Information Sheet and Research Electronic Data Capture Survey Data Dictionary [file jamanetwopen-e2239068-s001.pdf]

Supplementary Online Content

Singer J, Daum C, Shen MJ, et al. Assessment of oncology advanced practice professional willingness to participate in medical aid in dying. *JAMA Netw Open*. 2022;5(10):e2239068. doi:10.1001/jamanetworkopen.2022.39068

eAppendix. Study Information Sheet and Research Electronic Data Capture Survey Data Dictionary

This supplementary material has been provided by the authors to give readers additional information about their work.

© 2022 Singer J et al. *JAMA Network Open*.

eAppendix. Study Information Sheet and Research Electronic Data Capture Survey Data Dictionary

The Study Information Sheet (SIS) includes information provided to research subjects prior to consenting to the survey. The Research Electronic Data Capture (REDCap) survey includes underlying branching logic so not every question was asked of all participants.

© 2022 Singer J et al. *JAMA Network Open*.

Study Information Sheet (SIS)

IRO REC'D JUN 08 2021

FRED HUTCH
CURES START HERE®

Study Information Sheet

Advance Practice Providers and Washington State's Death with Dignity Act

Recently the Washington State legislature considered a bill that would have allowed Advance Practice Providers (APPs) to participate in Death with Dignity (DWD) as attending or consulting clinicians. This means that APPs would be able to prescribe a lethal dose of medication to adult patients with a terminal illness when that patient is expected to have a life expectancy of 6 months or less if the illness takes its usual course. As a result of this potential change in the law, we are interested in the perspectives of APPs on DWD.

This is a voluntary, on-line survey conducted via REDCap. You may elect to participate or opt-out. Your decision to opt-in or opt-out will not impact your employment or your relationship to your employer.

The survey should take 10-15 minutes to complete. You may skip any questions you prefer not to answer.

There are no costs or benefits to you if you participate. There is no payment for participation.

The risks of participation are primarily of breach of confidentiality. We will do everything we can to prevent this, including using study identifiers (e.g. instead of your name) and password-protected study files. While the biostatistician on this study will have access to identifiable data, researchers will only have access to de-identified data. Research results will only be presented in aggregate.

At the end of the survey you will be asked whether you are willing to be contacted in the future for additional studies.

If you participate in the survey and later decide that you do not want your information included, please email:

Elizabeth Loggers MD, PhD
Associate Member, Clinical Research Division, Fred Hutch
206.667.7442
eloggers@fredhutch.org
1100 Fairview Ave. N., Mail Stop D5-380
Seattle, WA 98109

FHCRC IRB Approval
07/01/2021
Document Released Date

© 2022 Singer J et al. *JAMA Network Open*.

Data Dictionary Codebook

09/09/2022 10:30pm

#	Variable / Field Name	Field Label<br>*Field Note*	Field Attributes (Field Type, Validation, Choices, Calculations, etc.)
**Instrument: Information Sheet (information_sheet) Enabled as survey**			
1	[record_id]	Record ID	text<br>Field Annotation: @HIDDEN
2	[informationsheet]	Section Header: *Advance Practice Providers and Washington State's Death with Dignity Act*<br>Recently the Washington State legislature considered a bill that would have allowed Advance Practice Providers (APPs) to participate in Death with Dignity (DWD) as attending or consulting clinicians. This means that APPs would be able to prescribe a lethal dose of medication to adult patients with a terminal illness when that patient is expected to have a life expectancy of 6 months or less if the illness takes its usual course. As a result of this potential change in the law, we are interested in the perspectives of APPs on DWD. This is a voluntary, on-line survey conducted via REDCap. You may elect to participate or opt-out. Your decision to opt-in or opt-out will not impact your employment or your relationship to your employer. The survey should take 10-15 minutes to complete. You may skip any questions you prefer not to answer. There are no costs or benefits to you if you participate. There is no payment for participation. The risks of participation are primarily of breach of confidentiality. We will do everything we can to prevent this, including using study identifiers (e.g. instead of your name) and password-protected study files. While the biostatistician on this study will have access to identifiable data, researchers will only have access to de-identified data. Research results will only be presented in aggregate. At the end of the survey you will be asked whether you are willing to be contacted in the future for additional studies. If you participate in the survey and later decide that you do not want your information included, please email: Elizabeth Loggers MD, PhD Associate Member, Clinical Research Division, Fred Hutch 206.667.7442 eloggers@fredhutch.org 1100 Fairview Ave. N., Mail Stop D5-380 Seattle, WA 98109	descriptive
3	[optinout]	By opting-in, you acknowledge you've read the study information sheet and are willing to participate in this 10-15 minute survey. Please opt-in or opt-out of this research study	radio, Required<br>1 Opt-in<br>2 Opt-out
4	[yes]<br>Show the field ONLY if:<br>[optinout] = '1'	You've decided to opt-in to taking the survey. Thank you! Please click submit to continue on to the survey.	descriptive
5	[no]<br>Show the field ONLY if:<br>[optinout] = '2'	You've decided to opt-out of the survey, and we will no longer contact you. If you change your mind, please contact Dr. Elizabeth Loggers (eloggers@fredhutch.org). Thank you for your time!	descriptive

6	[information_sheet_complete]	Section Header: *Form Status*<br>Complete?	dropdown<br>0 Incomplete<br>1 Unverified<br>2 Complete
**Instrument: Demographics (demographics)** Enabled as survey			
7	[age]	Section Header: *Demographics*<br>What is your age in years?	text (number), Identifier
8	[gender]	Which term(s) best express(es) your gender identity (that you wish to disclose)?Please select as many as applicable	checkbox<br>1 gender___1 Man<br>2 gender___2 Woman<br>3 gender___3 Genderfluid/Non-binary<br>4 gender___4 Transgender<br>5 gender___5 Prefer not to answer<br>6 gender___6 None or some of the above, I'd like to describe myself
9	[gender_other]<br>Show the field ONLY if: [gender(6)] = "1"	How would you best describe your gender expression?	text
10	[race]	What is your race/ethnicity? Please select as many as applicable	checkbox<br>1 race___1 American Indian or Alaska Native<br>2 race___2 Asian<br>3 race___3 Black or African American<br>4 race___4 Hispanic or Latinx<br>5 race___5 Native Hawaiian or Pacific Islander<br>6 race___6 White<br>88 race___88 Other<br>99 race___99 Prefer not to answer
11	[race_other_1]<br>Show the field ONLY if: [race(88)]='1'	If other, please describe your racial background	text
12	[english]	Do you speak, read, or write in a language(s) other than English?	yesno<br>1 Yes<br>0 No
13	[english_other]<br>Show the field ONLY if: [english]='1'	What other language(s) do you speak, read, or write in? Please list as many as applicable	text
14	[religion]	With which of the following do you identify as:Please select as many as applicable	checkbox<br>1 religion___1 Christian<br>2 religion___2 Muslim<br>3 religion___3 Buddhist<br>4 religion___4 Hindu<br>5 religion___5 Jewish<br>6 religion___6 Spiritual<br>7 religion___7 Agnostic<br>8 religion___8 Atheist/Non-religious<br>88 religion___88 None<br>99 religion___99 Other
15	[religion_other]<br>Show the field ONLY if: [religion(99)] = '1'	If you selected 'other', please specify here:	text

© 2022 Singer J et al. *JAMA Network Open*.

16	[religion_identify]	Which of the following most accurately reflects your spirituality?	radio<br>1 Religious, but not spiritual<br>2 Spiritual, but not religious<br>3 Neither spiritual nor religious<br>4 Both spiritual and religious
17	[religion_frequency]<br>Show the field ONLY if:<br>[religion(1)] = '1' OR [religion(2)] = '1' OR [religion(3)] = '1' OR [religion(4)] = '1' OR [religion(5)] = '1' OR [religion(6)] = '1' OR [religion(7)] = '1' OR [religion(99)] = '1'	How often do you engage in religious or spiritual activities (as you define them)?	radio<br>0 Not at all<br>1 Rarely<br>2 Yearly<br>3 Monthly<br>4 Weekly<br>5 Daily
18	[political_interest]	How interested are you in politics, government, and public affairs?	radio<br>1 Not at all interested<br>2 Slightly interested<br>3 Somewhat interested<br>4 Interested<br>5 Very interested
19	[role]	Section Header: *Education/Professional Experience*<br>What is your role? Please select as many as applicable	checkbox<br>1 role___1 PA-C<br>2 role___2 ARNP<br>88 role___88 Other
20	[role_other]<br>Show the field ONLY if:<br>[role(88)] = '1'	If your role is not listed above, please specify here:	text
21	[years_practice]	How many years have you been practicing as an APP?	radio<br>1 0-2 years<br>2 3-5 years<br>3 6-10 years<br>4 11-20 years<br>5 >20 years
22	[years_practice_2]	How many years have you been practicing as an APP in Washington State?	radio<br>1 0-2 years<br>2 3-5 years<br>3 6-10 years<br>4 11-20 years<br>5 >20 years
23	[years_practice_3]	How many years have you been practicing as an APP in Oncology?	radio<br>1 0-2 years<br>2 3-5 years<br>3 6-10 years<br>4 11-20 years<br>5 >20 years
24	[practicetype]	Where you do currently pratice as an APP? Please select as many as applicable	checkbox<br>1 practicetype___1 Inpatient<br>2 practicetype___2 Outpatient

© 2022 Singer J et al. *JAMA Network Open*.

25	[field]	What field do you currently practice in?	radio<br>1 Hematology/Malignant Hematology<br>2 Transplant<br>3 Immunotherapy<br>4 Medical Oncology<br>5 Radiation Oncology<br>6 Surgical Oncology<br>7 Supportive Care<br>99 Other
26	[field_other]<br>Show the field ONLY if:<br>[field] = '99'	If you practice in another field, please specify here:	text
27	[demographics_complete]	Section Header: *Form Status*<br>Complete?	dropdown<br>0 Incomplete<br>1 Unverified<br>2 Complete

Instrument: Maidquestions (maidquestions) **Enabled as survey**

28	[personal_seriousill]	Section Header: *Prior Knowledge*<br>Do you have any personal experience with serious illness?	radio<br>1 Yes, my own illness<br>2 Yes, my loved one/close friend/family<br>3 Both<br>4 Neither
29	[personal_dwd]	Do you have any personal experience with Death with Dignity?	radio<br>1 Yes, my loved one/friend/family<br>2 Yes, prior professional experience<br>3 Both<br>4 Neither
30	[dwd_aware]	Prior to this survey, were you aware that Death with Dignity was legal in Washington State?	radio<br>1 Yes, fully aware<br>2 Yes, somewhat aware<br>3 No, not aware
31	[dwd_prior_education]<br>Show the field ONLY if:<br>[dwd_aware] = '1' OR [dwd_aware] = '2'	Have you ever participated in any educational activities (e.g., grand rounds; didactics) about Death with Dignity?	yesno<br>1 Yes<br>0 No

© 2022 Singer J et al. *JAMA Network Open*.

32	[training_topic]	Given that the proposed legislation would have allowed APP's to participate as prescribing or consulting clinicians in Death with Dignity, what training do you think APP's would need, if any? Select all that apply	checkbox<br>1 | training_topic___1 | General information about Death with Dignity process<br>2 | training_topic___2 | Assessing eligibility<br>3 | training_topic___3 | Estimating prognosis<br>4 | training_topic___4 | Educating the patient (and/or family)<br>5 | training_topic___5 | Responding to patient requests<br>6 | training_topic___6 | Consulting clinician responsibilities<br>7 | training_topic___7 | Prescribing clinician responsibilities<br>8 | training_topic___8 | Knowledge about Death with Dignity medications<br>9 | training_topic___9 | Education regarding meeting legal requirements (i.e. waiting period, forms)<br>88 | training_topic___88 | Other<br>99 | training_topic___99 | None
33	[support]	What type of training do you think would be useful? Select all that apply	checkbox<br>1 | support___1 | Group Training<br>2 | support___2 | 1:1 Training<br>3 | support___3 | Shadowing<br>4 | support___4 | Didactic (Reading, Lectures)<br>88 | support___88 | Other<br>99 | support___99 | None
34	[support_other]<br>Show the field ONLY if:<br>[support(88)] = '1'	If another kind of support is needed, please specify:	text
35	[planning]	If it were legal for APP's to participate as consulting or prescribing clinicians, would you plan on participating in Death with Dignity, in any capacity?	radio<br>1 | Yes, as consulting and prescribing<br>2 | Yes, as consulting but not prescribing<br>3 | Unsure<br>4 | No
36	[planning_why]<br>Show the field ONLY if:<br>[planning] = '1' OR [planning] = '2' OR [planning] = '3' OR [planning] = '4'	Please tell us why:	notes
37	[comfort_1]	Section Header: *Please respond to the question: "In regards to Death with Dignity, how comfortable do you feel about..."*<br>Advanced Practice Providers proposed role in Death with Dignity?	radio (Matrix)<br>1 | Very Uncomfortable<br>2 | Uncomfortable<br>3 | Somewhat Comfortable<br>4 | Comfortable<br>5 | Very Comfortable

© 2022 Singer J et al. *JAMA Network Open*.

38	[comfort_2]	Responding to patient requests for Death with Dignity?	radio (Matrix)<br>1 Very Uncomfortable<br>2 Uncomfortable<br>3 Somewhat Comfortable<br>4 Comfortable<br>5 Very Comfortable
39	[comfort_3]	Raising Death with Dignity as a possible option for your patients?	radio (Matrix)<br>1 Very Uncomfortable<br>2 Uncomfortable<br>3 Somewhat Comfortable<br>4 Comfortable<br>5 Very Comfortable
40	[comfort_4]	Defining prognosis?	radio (Matrix)<br>1 Very Uncomfortable<br>2 Uncomfortable<br>3 Somewhat Comfortable<br>4 Comfortable<br>5 Very Comfortable
41	[comfort_6]	Acting as the consulting clinician?	radio (Matrix)<br>1 Very Uncomfortable<br>2 Uncomfortable<br>3 Somewhat Comfortable<br>4 Comfortable<br>5 Very Comfortable
42	[comfort_7]	Prescribing Death With Dignity medications?	radio (Matrix)<br>1 Very Uncomfortable<br>2 Uncomfortable<br>3 Somewhat Comfortable<br>4 Comfortable<br>5 Very Comfortable
43	[comfort_8]	Discussing with family and loved ones the patient's decision to pursue Death with Dignity?	radio (Matrix)<br>1 Very Uncomfortable<br>2 Uncomfortable<br>3 Somewhat Comfortable<br>4 Comfortable<br>5 Very Comfortable
44	[knowledge_1]	Section Header: *Please respond to the question: "In regards to Death with Dignity, how knowledgeable do you feel about..."*<br>Assessing the eligibility of a patient?	radio (Matrix)<br>1 Not at All Knowledgeable<br>2 Not Very Knowledgeable<br>3 Somewhat Knowledgeable<br>4 Knowledgeable<br>5 Very Knowledgeable
45	[knowledge_2]	Defining prognosis?	radio (Matrix)<br>1 Not at All Knowledgeable<br>2 Not Very Knowledgeable<br>3 Somewhat Knowledgeable<br>4 Knowledgeable<br>5 Very Knowledgeable

46	[knowledge_3]	Educating the patient (and/or their family)?	radio (Matrix)<br>1 Not at All Knowledgeable<br>2 Not Very Knowledgeable<br>3 Somewhat Knowledgeable<br>4 Knowledgeable<br>5 Very Knowledgeable
47	[knowledge_4]	Acting as the consulting clinician?	radio (Matrix)<br>1 Not at All Knowledgeable<br>2 Not Very Knowledgeable<br>3 Somewhat Knowledgeable<br>4 Knowledgeable<br>5 Very Knowledgeable
48	[knowledge_5]	Prescribing Death With Dignity medications?	radio (Matrix)<br>1 Not at All Knowledgeable<br>2 Not Very Knowledgeable<br>3 Somewhat Knowledgeable<br>4 Knowledgeable<br>5 Very Knowledgeable
49	[knowledge_7]	Accurately following the legal requirements (e.g. waiting period, forms)	radio (Matrix)<br>1 Not at All Knowledgeable<br>2 Not Very Knowledgeable<br>3 Somewhat Knowledgeable<br>4 Knowledgeable<br>5 Very Knowledgeable
50	[views_1]	Section Header: *How much do the following influence your views on Death with Dignity?*<br>Political Beliefs	radio (Matrix)<br>1 Not at all Influential<br>2 Slightly Influential<br>3 Somewhat Influential<br>4 Very Influential<br>5 Extremely Influential
51	[views_2]	Religious Beliefs	radio (Matrix)<br>1 Not at all Influential<br>2 Slightly Influential<br>3 Somewhat Influential<br>4 Very Influential<br>5 Extremely Influential
52	[views_3]	Individual Values	radio (Matrix)<br>1 Not at all Influential<br>2 Slightly Influential<br>3 Somewhat Influential<br>4 Very Influential<br>5 Extremely Influential
53	[views_4]	Family Values	radio (Matrix)<br>1 Not at all Influential<br>2 Slightly Influential<br>3 Somewhat Influential<br>4 Very Influential<br>5 Extremely Influential

© 2022 Singer J et al. *JAMA Network Open*.

54	[views_5]	Personal Experience	radio (Matrix)<br>1 Not at all Influential<br>2 Slightly Influential<br>3 Somewhat Influential<br>4 Very Influential<br>5 Extremely Influential
55	[views_6]	Professional Experience	radio (Matrix)<br>1 Not at all Influential<br>2 Slightly Influential<br>3 Somewhat Influential<br>4 Very Influential<br>5 Extremely Influential
56	[views_7]	Educational Experience	radio (Matrix)<br>1 Not at all Influential<br>2 Slightly Influential<br>3 Somewhat Influential<br>4 Very Influential<br>5 Extremely Influential
57	[dwd_involvement_number]	Section Header: *In this section, we'll ask you a few questions about your experience with patients who inquire about or request Death with Dignity*<br>How many of your patient(s) have inquired or have you spoken to about Death with Dignity?	radio (Matrix)<br>1 None<br>2 1-3 patients<br>3 4-10 patients<br>4 11-20 patients<br>5 21-40 patients<br>6 >40 patients
58	[dwd_involvement_number_2]	How many of your patient(s) have pursued Death with Dignity?	radio (Matrix)<br>1 None<br>2 1-3 patients<br>3 4-10 patients<br>4 11-20 patients<br>5 21-40 patients<br>6 >40 patients

59	[dwd_involvement_no_why]<br>Show the field ONLY if:<br>[dwd_involvement_number_2] = '1'	If you have yet to work with a patient pursuing Death with Dignity, why is that?Please select as many as applicable	checkbox<br>1 dwd_involvement_no_why___1 Personal morals and values<br>2 dwd_involvement_no_why___2 Religious Beliefs<br>3 dwd_involvement_no_why___3 Political Beliefs<br>4 dwd_involvement_no_why___4 Lack of knowledge with Death with Dignity process<br>5 dwd_involvement_no_why___5 Discomfort due to specifics of patient and/or family<br>6 dwd_involvement_no_why___6 Discomfort due to prior professional experience<br>7 dwd_involvement_no_why___7 Discomfort due to prior personal experience<br>8 dwd_involvement_no_why___8 No opportunity to do so<br>99 dwd_involvement_no_why___99 Other
60	[dwd_involvement_no_why_other]<br>Show the field ONLY if:<br>[dwd_involvement_no_why(99)] = '1'	If another reason, please specify:	text
61	[dwd_yes_1]<br>Show the field ONLY if:<br>[dwd_involvement_number_2] = '3' OR [dwd_involvement_number_2] = '4' OR [dwd_involvement_number_2] = '5' OR [dwd_involvement_number_2] = '6'	Section Header: *In this section, we'll ask you a few questions about the pathways taken by patients who have requested Death with Dignity*<br>Have you ever been involved with a patient who requested the Death with Dignity process, but ended up being ineligible?	radio (Matrix)<br>1 Yes<br>0 No
62	[dwd_yes_2]<br>Show the field ONLY if:<br>[dwd_involvement_number_2] = '3' OR [dwd_involvement_number_2] = '4' OR [dwd_involvement_number_2] = '5' OR [dwd_involvement_number_2] = '6'	Have you ever worked with a patient who requested the Death with Dignity process, but changed their mind about proceeding?	radio (Matrix)<br>1 Yes<br>0 No
63	[dwd_yes_3]<br>Show the field ONLY if:<br>[dwd_involvement_number_2] = '3' OR [dwd_involvement_number_2] = '4' OR [dwd_involvement_number_2] = '5' OR [dwd_involvement_number_2] = '6'	Have you ever discussed Death with Dignity with a patient's family member or loved one?	radio (Matrix)<br>1 Yes<br>0 No

© 2022 Singer J et al. *JAMA Network Open*.

64	[dwd_yes_4]<br><br>Show the field ONLY if: [dwd_involvement_number_2] = '3' OR [dwd_involvement_number_2] = '4' OR [dwd_involvement_number_2] = '5' OR [dwd_involvement_number_2] = '6'	Have you ever worked with a Death with Dignity patient who acquired the medication?	radio (Matrix)<br>1 Yes<br>0 No
65	[dwd_yes_5]<br><br>Show the field ONLY if: [dwd_involvement_number_2] = '3' OR [dwd_involvement_number_2] = '4' OR [dwd_involvement_number_2] = '5' OR [dwd_involvement_number_2] = '6'	Have you ever worked with a Death with Dignity patient who ingested the medication to end their life?	radio (Matrix)<br>1 Yes<br>0 No
66	[ineligible]<br><br>Show the field ONLY if: [dwd_yes_1] = '1'	If you've had a patient(s) who pursued Death with Dignity but became ineligibile, choose all that apply: If you've had more than one Death with Dignity ineligible patient, please choose as many as applicable across all Death with Dignity patients you've worked with	checkbox<br>1 ineligible___1 Too Sick/Progressed Disease<br>2 ineligible___2 Absorption Issues<br>3 ineligible___3 Mental Competency<br>4 ineligible___4 Inability to Self-Administer<br>5 ineligible___5 Cost of Medication<br>88 ineligible___88 Other
67	[ineligible_other]<br><br>Show the field ONLY if: [ineligible(88)] = '1'	If the patient became ineligible for another reason, please specify:	text
68	[declined]<br><br>Show the field ONLY if: [dwd_yes_2] = '1'	If you've had a patient(s) who pursued Death with Dignity but declined proceeding, choose all apply:If you've had more than one Death with Dignity patient who declined proceeding, please choose as many as applicable across all Death with Dignity patients you've worked with	checkbox<br>1 declined___1 Cost of or access to medication<br>2 declined___2 Patient reassessed their values<br>3 declined___3 Patient not supported by family<br>4 declined___4 Patient concerned of stigma on self or family<br>5 declined___5 Pursued other options (e.g. palliative care, hospice)<br>88 declined___88 Other
69	[declined_other]<br><br>Show the field ONLY if: [declined(88)] = '1'	If the patient declined proceeding for another reason, please specify:	text
70	[experience_1]<br><br>Show the field ONLY if: [dwd_involvement_number_2] = '3' OR [dwd_involvement_number_2] = '4' OR [dwd_involvement_number_2] = '5' OR [dwd_involvement_number_2] = '6'	How have your relationships with patients been affected by their pursuit of Death with Dignity?	radio<br>1 Very Positive<br>2 Mostly Positive<br>3 Neutral<br>4 Mostly Negative<br>5 Very Negative<br>6 Varied, depends on the case
71	[experience_4]<br><br>Show the field ONLY if: [dwd_involvement_number_2] = '3' OR [dwd_involvement_number_2] = '4' OR [dwd_involvement_number_2] = '5' OR [dwd_involvement_number_2] = '6'	How have your relationships with patient's family members been affected by the patient's pursuit of Death with Dignity?	radio<br>1 Very Positive<br>2 Mostly Positive<br>3 Neutral<br>4 Mostly Negative<br>5 Very Negative<br>6 Varied, depends on the case

© 2022 Singer J et al. *JAMA Network Open*.

72	[experience_2]<br><br>Show the field ONLY if:<br>[dwd_involvement_number_2] = '3' OR [dwd_involvement_number_2] = '4' OR [dwd_involvement_number_2] = '5' OR [dwd_involvement_number_2] = '6'	How has your relationships with fellow APP's been affected by your invovlement in the Death with Dignity process?	radio<br>1 Very Positive<br>2 Mostly Positive<br>3 Neutral<br>4 Mostly Negative<br>5 Very Negative<br>6 Varied, depends on the case
73	[experience_3]<br><br>Show the field ONLY if:<br>[dwd_involvement_number_2] = '3' OR [dwd_involvement_number_2] = '4' OR [dwd_involvement_number_2] = '5' OR [dwd_involvement_number_2] = '6'	How has your relationships with attendings been affected by your invovlement in the Death with Dignity process?	radio<br>1 Very Positive<br>2 Mostly Positive<br>3 Neutral<br>4 Mostly Negative<br>5 Very Negative<br>6 Varied, depends on the case
74	[maidquestions_complete]	Section Header: *Form Status*<br>Complete?	dropdown<br>0 Incomplete<br>1 Unverified<br>2 Complete
**Instrument: Healthcare (healthcare)** Enabled as survey			
75	[dwd_legal_1]	Section Header: *In this section, we are curious about your perceptions on the legality of Death with Dignity*<br>Do you think Death with Dignity should be legal?	radio (Matrix)<br>1 Strongly Disagree<br>2 Disagree<br>3 Neutral<br>4 Agree<br>5 Strongly Agree
76	[dwd_legal_2]	Do you think advanced practice providers should be able to act as a consulting clinician for Death with Dignity?	radio (Matrix)<br>1 Strongly Disagree<br>2 Disagree<br>3 Neutral<br>4 Agree<br>5 Strongly Agree
77	[dwd_legal_3]	Do you think advanced practice providers should be able to act as an attending/prescribing clinician for Death with Dignity?	radio (Matrix)<br>1 Strongly Disagree<br>2 Disagree<br>3 Neutral<br>4 Agree<br>5 Strongly Agree
78	[dwd_option_1]	Do you think Death with Dignity should be introduced by providers as option for patients with terminal illness?	radio (Matrix)<br>1 Strongly Disagree<br>2 Disagree<br>3 Neutral<br>4 Agree<br>5 Strongly Agree

© 2022 Singer J et al. *JAMA Network Open*.

79	[dwd_legal_length]	What should the waiting period be to recieve the medications?	radio<br>1 None<br>2 24 hours<br>3 72 hours<br>4 7 days<br>5 14 days<br>6 >/= 21 days<br>7 Unsure
80	[dwd_legal_length_2]	What should the prognosis be for patients with a terminal illness to be eligible to participate in Death with Dignity?	radio<br>1 None<br>2 < 72 hours<br>3 < 7 days<br>4 < 14 days<br>5 < 1 month<br>6 < 3 months<br>7 < 6 months<br>8 < 1 year<br>9 > 1 year<br>10 Unsure
81	[interview_yesno]<br>Show the field ONLY if: [dwd_aware] = '1' OR [dwd_aware] = '2'	Would you be willing to participate in an interview or a focus group on Death with Dignity?	yesno<br>1 Yes<br>0 No
82	[consenting]	Could we contact you in the future about other studies related to this topic?	yesno<br>1 Yes<br>0 No
83	[healthcare_complete]	Section Header: *Form Status*<br>Complete?	dropdown<br>0 Incomplete<br>1 Unverified<br>2 Complete

© 2022 Singer J et al. *JAMA Network Open*.
